# Stem Cells from Human Exfoliated Deciduous Teeth (SHEDs) and Dental Pulp Stem Cells (DPSCs) Display a Similar Profile with Pericytes

**DOI:** 10.1155/2021/8859902

**Published:** 2021-07-24

**Authors:** Shao Yue Zhu, Chang Yong Yuan, Yi Fan Lin, Hao Liu, Yan Qi Yang, Hai Ming Wong, Cheng Fei Zhang, Peng Lai Wang, Min Gu

**Affiliations:** ^1^Discipline of Paediatric Dentistry and Orthodontics, Faculty of Dentistry, The University of Hong Kong, Hong Kong, China; ^2^Orthodontic Department, Affiliated Stomatological Hospital of Xuzhou Medical University, Xuzhou, China; ^3^Discipline of Oral and Maxillofacial Surgery, Affiliated Stomatological Hospital of Xuzhou Medical University, Xuzhou, China; ^4^Endodontology, Faculty of Dentistry, The University of Hong Kong, Hong Kong, China; ^5^Dental Implant Center, Affiliated Stomatological Hospital of Xuzhou Medical University, Xuzhou, China

## Abstract

**Background:**

Pericytes play an important role in forming functional blood vessels and establishing stable and effective microcirculation, which is crucial for vascular tissue engineering. The slow ex vivo expansion rate, limited proliferative capacity, and variability of tissue-specific phenotypes would hinder experimental studies and clinical translation of primary pericytes. In this study, the angiogenic and pericyte functions of stem cells from human exfoliated deciduous teeth (SHEDs) and postnatal human dental pulp stem cells (DPSCs) were investigated.

**Methods:**

Osteogenic and adipogenic induction assays were performed to evaluate the mesenchymal potential of SHEDs, DPSCs, and pericytes. An in vitro Matrigel angiogenesis assay was conducted to reveal the ability of SHEDs, DPSCs, and pericytes to stabilize vascular-like structures. Quantitative real-time polymerase chain reaction (RT-qPCR) was performed to evaluate mRNA expression. Flow cytometry, western blotting, and immunostaining were used to assess the protein expression. Wound healing and transwell assays were performed to evaluate the migration ability of SHEDs, DPSCs, and pericytes.

**Results:**

The osteogenic and adipogenic induction assays showed that SHEDs, DPSCs, and pericytes exhibited similar stem cell characteristics. The mRNA expression levels of PDGFR-*β*, *α*-SMA, NG2, and DEMSIN in SHEDs and DPSCs cultured in EC medium were significantly higher than those in the control groups on day 7 (*P* < 0.05), but significantly higher than those in the pericytes group on day 14 (*P* < 0.05). Flow cytometry showed that high proportions of SHEDs and DPSCs were positive for various pericyte markers on day 7. The DPSCs, SHEDs, and pericytes displayed strong migration ability; however, there was no significant difference among the groups (*P* > 0.05).

**Conclusion:**

The SHEDs and DPSCs display a profile similar to that of pericytes. Our study lays a solid theoretical foundation for the clinical use of dental pulp stem cells as a potential candidate to replace pericytes.

## 1. Introduction

As a key cellular component of vascular structures, pericytes play a critical role in skeletal muscle regeneration, angiogenesis, in the survival of endothelial cells, c fat formation, and blood–brain barrier, and blood flow. Pericytes are embedded in the basement membrane where they communicate with endothelial cells of the body's smallest blood vessels by means of both direct physical contact and paracrine signaling [1]. Previous studies have strongly indicated a fascinating interaction and interdependence between pericytes and endothelial cells (ECs) [2]. In addition, pericytes, which allow endothelial cells to differentiate, multiply, and form vascular branches [3], play a crucial role in the formation and functionality of the selectively permeable space between the circulatory system and central nervous system [4], regulating blood flow at the capillary level [5]. All the advantages mentioned above make them promising candidates for tissue engineering. Nevertheless, the slow ex vivo expansion rate, limited proliferative capacity, and variability of tissue-specific phenotypes would hinder experimental studies and the clinical translation of primary pericytes [6]. Hence, it would be beneficial to find an alternative cell source for pericytes. In the past few years, few cell types have been used as mesenchymal stem cells (MSCs) [7]. Whether or not MSCs are pericytes have always been an interesting but confusing question. By comparing pericytes with MSCs from different tissues, some researchers proposed a hypothesis that pericytes and MSCs are similar cells residing in the wall of the vasculature [8]. However, it is very difficult to draw definite conclusions. Blocki et al. [6] used functional assays such as spheroid sprouting and Matrigel assays to compare the angiogenic abilities of two different types of MSCs aspirated from human fresh bone marrow, and the results showed that only one type of MSCs could function as pericytes. The exploration of different tissues for MSC-like cells has resulted in the discovery of many different tissue-derived MSC populations such as dental-specific MSC-like populations, including postnatal human dental pulp stem cells (DPSCs) [9], stem cells from human exfoliated deciduous teeth (SHED) [10], periodontal ligament stem cells (PDLSCs) [11], stem cells from apical papilla (SCAP) [12], and dental follicle stem cells (DFPCs) [13]. Dental pulp stem cells can be readily harvested from exfoliated deciduous or wisdom teeth, without imposing any additional discomfort or injury to patient donors, making them the most promising candidates for tissue engineering related to stem cells. Considering the MSC-like properties, the relationship between dental pulp stem cells and pericytes has been the focus of studies. A study performed by Janebodin et al. [14] showed that DPSCs could function as angiogenic- and pericyte-inducing cells. In our recent in vitro and in vivo studies, we successfully fabricated prevascularized tissue-engineered pulp constructs by seeding ECs with DPSCs [15]. Although these studies have been encouraging, no study has directly and systematically compared dental stem cells with pericytes.

In this study, we aimed to investigate the angiogenic and pericyte functions of SHEDs and DPSCs. We chose angiogenesis capacity (including the vessel stabilization ability, contractile capacity, transport, and capillary permeability regulation ability, as well as the capacity to govern angiogenic or quiescent blood vessels), macrophage-like properties, and mesenchymal potential as evaluation standards to compare the similarity among DPSCs, SHEDs, and pericytes. Using rigorous standard methodology, we aimed to determine if DPSCs and SHEDs share similar functions with pericytes.

## 2. Methods

### 2.1. Cell Culture

The SHEDs were purchased from ALLCELLS (Alameda, CA, USA), and the DPSCs were provided by Songtao Shi (Department of Anatomy and Cell Biology, University of Pennsylvania School of Dental Medicine), and their use was approved by the Ethics Committee of the University of Pennsylvania. Stem cell phenotypic markers, CD45, CD90, CD105, and CD73, were used to evaluate SHED and DPSC stemness using flow cytometry.

Human brain vascular pericytes (cat. #1200) and human umbilical vein endothelial cells (HUVECs) (Cat. #8000) were purchased from ScienCell (Carlsbad, CA, USA) and were cultured in Pericyte Medium (PM, ScienCell) and fully supplemented with endothelial growth medium (EGM-2, Lonza Walkersville, MD, USA), respectively.

### 2.2. Mesenchymal Differentiation

We followed our previously published study on mesenchymal differentiation [16]. For osteogenic differentiation, pericytes, SHED, and DPSCs were seeded onto 12-well plates at a concentration of 4 × 10^3^ cells/cm^2^ and cultured to 70% confluence. They were then induced in Dulbecco's Modified Eagle Medium (DMEM) (DMEM, ScienCell) supplemented with 10% fetal bovine serum (FBS), 1% penicillin/streptomycin, 50 *μ*g/mL L-ascorbic acid phosphate, 10 mmol/L *β*-glycerophosphate, 10 nmol/L dexamethasone, and 10 nmol/L 1,25 dihydroxyvitamin D3 for 4 weeks, with the medium changed every 3 d. After 4 weeks of induction, the samples were fixed with 10% neutral-buffered formalin for 1 h and rinsed with distilled H_2_O. Mineral staining was performed using an Alizarin Red S (Sigma-Aldrich, St. Louis, MO, USA) staining solution.

For adipogenic differentiation, pericytes, SHED, and DPSCs were seeded onto 12-well plates at a concentration of 4 × 10^3^ cells/cm^2^ and cultured to 70% confluence and, then, inducted in DMEM supplemented with 10% FBS, 1% penicillin/streptomycin, 1 *μ*g/mL insulin, 1 *μ*mol/L dexamethasone, and 0.5 mmol/L 3-isobutyl-1-methylxanthine (IBMX) for 4 weeks, with medium changed every 3 d. After 4 weeks of induction, the samples were fixed with 10% neutral-buffered formalin for 1 h and rinsed with 70% ethanol. Lipid droplets were stained with 0.21% Oil red O (Sigma-Aldrich) staining solution. Neurogenic differentiation capacity was tested using neurogenic induction medium (Neurobasal A [Gibco-Invitrogen]) combined with 40 ng/mL fibroblast growth factor (BD Biosciences, Bedford, MA, USA) and 20 ng/mL epidermal growth factor (BD Biosciences). Immunofluorescence staining of *β*III-tubulin was conducted after 4 weeks of induction.

### 2.3. Flow Cytometry Analysis

Flow cytometry was performed as previously described using the BD Cytofix/Cytoperm™ Fixation/Permeabilization Kit (554714; BD Biosciences) to detect the expression of the various aforementioned smooth muscle cell markers by SHED-derived smooth muscle cells (SMCs). A cell dissociation solution (C5914; Sigma-Aldrich) was used to disperse the SHED-derived SMCs. After fixation and permeabilization, cells were incubated for 2 h with primary antibodies against *α*-smooth muscle actin (*α*-SMA) (ab124964; Abcam, Cambridge, MA, USA), SM22 alpha (ab14106; Abcam), smooth muscle myosin heavy chain 11 (ab683; Abcam), and calponin (ab46794; Abcam), and then washed with phosphate-buffered saline (PBS). This was followed by incubation with the appropriate secondary antibodies: goat anti-mouse IgG H&L (Alexa Fluor® 488) preadsorbed (ab150117; Abcam) and goat anti-rabbit IgG H&L (Alexa Fluor® 488). In this study, mouse IgG1, kappa monoclonal (ab170190; Abcam), and rabbit IgG monoclonal [EPR25A] (ab172730; Abcam) were used as isotype controls. Flow cytometry data were analyzed using the FACSVerse software (BD Biosciences).

### 2.4. In Vitro Matrigelangiogenesis Assay

To investigate whether pericytes, SHEDs, and DPSCs have a similar ability to promote the stability of vascular structures, an *in vitro* Matrigel angiogenesis study was performed according to a published protocol [17]. Briefly, 3.6 × 10^4^ primary HUVECs were seeded on one well of 48-well plates that had been precoated with Matrigel (354230; BD Biosciences) and incubated at 37°C and 5% CO_2_. When the typical vascular structures were formed (approximately 6 h from the seeding of HUVECs on Matrigel), different groups of cells (pericytes, SHED, and DPSC groups) were added at a density of 7200 cells per well. Images were captured every 6 h for over 3 days. For some applications, different fluorescent probes (Cell Tracker® Red/Green CMTPX Dye, Thermo Fisher Scientific, MA, USA) were used to label HUVECs and pericytes/SHED/DPSCs (red for HUVECs, and green for others). Images were captured under a confocal laser scanning microscope (FluoView™ FV1000, Olympus, Tokyo, Japan) and analyzed using the ImageJ software (National Institutes of Health, Bethesda, MD, USA).

### 2.5. Migratory Capacities of SHEDs, DPSCs, and Pericytes

#### 2.5.1. The Migratory Capacities of SHEDs, DPSCs, and Pericytes

The migratory capacities of SHEDs, DPSCs, and pericytes were assessed by transwell migration assays using Boyden chambers consisting of transwell filter inserts in 24-well tissue culture plates (BD LabWare, Bedfold, MA, USA). After 12 h of serum starvation, 1 × 10^5^ cells/well of SHEDs, DPSCs, or pericytes were cultured in the upper chamber. Viable cells on the lower membrane surface were counted at 6, 8, 10, and 12 h.

#### 2.5.2. The Influence of SCAPs, DPSCs, and Pericytes on the Migratory Behavior of HUVECs

The influence of SCAPs, DPSCs, and pericytes on the migratory behavior of HUVECs was assessed using a transwell migration assay. Briefly, each type of formal motioned cells and HUVECs were subjected to serum starvation for 24 h before the experiment. Subsequently, these cells were trypsinized and resuspended in a culture medium to a titer of 1 × 10^6^/mL. Within each transwell insert, 0.1 mL of the HUVEC suspension was seeded, whereas 0.6 mL of the SCAPs, DPSCs, or pericyte suspension were plated in the lower wells. Transwells were incubated for 24 h at 37°C in a 5% CO_2_ incubator. After that, the adherent cells at the bottom membrane of the inserts were counted at 6, 8, 10, and 12 h.

### 2.6. Wound Healing Assay

Migration of the derived human-induced pluripotent stem cell (hiPSC) perivascular cells was assessed using a wound-healing assay [18]. The cells were cultured in a confluent monolayer in a six-well plate. Cell monolayers were wounded by scratching a strip of cells with a 200-*μ*L pipette tip. After the detached cells were removed and the cells were washed, a fresh medium containing 0.5% serum was added. Cells were incubated in a humidified incubator coupled to a microscope, which took a series of images of the migration of the cells into the gap every 10 min for 24 h. Migration trajectories and speed were calculated using the MTrackJ plugin of ImageJ.

### 2.7. Real-Time Quantitative Reverse Transcription PCR Assay

To investigate the mRNA expression levels of NG2, DESMIN, *α*-SMA, and PDGFR-*β* in the SHEDs, DPSCs, and pericytes, these cells were cultured in *α*-Minimum Essential Medium (MEM), Pericyte Medium, and MesenCult™ MSC Basal Medium (STEMCELL Technologies, Vancouver, BC, Canada) supplemented with MesenCult™ MSC Stimulatory Supplement (Cat. 05402; STEMCELL) for 2 weeks, and total RNA was extracted with an RNeasy Plus Mini Kit (Qiagen, Hilden, Germany). Subsequently, 1.0 *μ*g of total RNA was reverse transcribed into cDNA in a total reaction mixture of 10 *μ*L using Super-Script VILO Master Mix (Invitrogen SuperScript™ III Reverse Transcriptase, Thermo Fisher Scientific). Real-time quantitative reverse transcription PCR (qRT-PCR) was performed using an ABI Prism 7000 Sequence Detection System (Applied Biosystems, Foster City, CA, USA) with SYBR Green reagent (Applied Biosystems). Glyceraldehyde 3-phosphate dehydrogenase (GAPDH) was used as an endogenous control. Standards and samples were run in triplicates. The primer sequences are listed in Table 1.

### 2.8. Western Blot Analysis

Western blot assays were performed to detect the protein expression levels of NG2, DESMIN, *α*-SMA, and PDGFR-*β* in the SHEDs, DPSCs, and pericytes. Briefly, cells were seeded at a density of 1 × 10^5^ cells per well in 60-mm plates, 10 mL of *α*-MEM, PM, or MesenCult™ MSC Basal Medium (STEMCELL Technologies) supplemented with MesenCult™ MSC Stimulatory Supplement (Cat. 05402; STEMCELL Technologies) was added and changed every 3 d for 2 weeks. Mammalian Protein Extraction Reagent (M-PER) protein extraction buffer containing 1x protease inhibitor cocktail (Thermo Fisher Scientific) was used to extract total protein. A BCA kit (Thermo Fisher Scientific) was used to quantify the protein concentrations. Samples were separated on a 10% polyacrylamide gel and transferred onto an ImmunBlot PVDF membrane (EMD Millipore, Billerica, MA, USA). Membranes were blocked with 5% milk in Tris-phosphate buffer containing 0.05% Tween-20 (TBS-T) for 1 h at room temperature and, then, incubated overnight with primary antibodies specific for NG2 (LS-C72310; LSBio, Seattle, WA, USA), DESMIN (ab15200; Abcam), PDGFR-*β* (ab69506; Abcam), *α*-SMA (ab124964; Abcam), and beta-actin (sc-47778; Santa Cruz Biotechnologies, California, USA) at 4°C. After three washes with TBS-T for 5 min each, membranes were incubated with horseradish peroxidase-conjugated anti-rabbit (7074; Cell Signaling Technology, MA, USA) or anti-mouse (7076; Cell Signaling Technology) secondary antibodies for 1 h and, then, washed three times with TBS-T. Blots were visualized and digitized using enhanced chemiluminescence (Thermo Fisher Scientific).

### 2.9. Immunocytochemistry Assay

The SHEDs, DPSCs, and pericytes were cultured in MesenCult™ MSC Basal Medium (Cat.05401; STEMCELL Technologies) supplemented with MesenCult™ MSC Stimulatory Supplement (Cat. 05402S; TEMCELL Technologies) for 2 weeks. Subsequently, the cells were fixed with 4% (*w*/*v*) cold paraformaldehyde (PFA) for 15 min and washed with PBS. The cells were then permeabilized using 0.1% (v/v) Triton-X100 (Sigma-Aldrich) for 10 min. Primary antibodies against NG2 (LS-C72310; LSBio, Seattle, WA, USA), DESMIN (ab15200; Abcam), and PDGFR-*β* (ab69506; Abcam) were used for immunocytochemical staining. Goat anti-rabbit IgG H&L (Alexa Fluor® 488) preadsorbed (ab150077; Abcam) and goat anti-mouse IgG H&L (Alexa Fluor® 488) (ab150117; Abcam) were used as secondary antibodies. The images were captured under fluorescence (Olympus BX60; Olympus, Center Valley, PA, USA) and confocal laser scanning microscopy (LSM 510-META; Carl Zeiss, Jena, Germany).

### 2.10. Statistical Analysis

Real-time RT-PCR, functionality assays, flow cytometry, and image analyses were performed in at least triplicate biological samples. Real-time RT-PCR analyses were performed in triplicate. Unpaired two-tailed *t*-tests, one-way analysis of variance (ANOVA), and Bonferroni posttests were performed using GraphPad Prism 4.02 (GraphPad Software Inc., La Jolla, CA, USA). Significance levels were set at ^∗^*p* < 0.05, ^∗∗^*p* < 0.01, and ^∗∗∗^*p* < 0.001. All graphical data are reported as mean ± standard error of the mean (SEM).

## 3. Results

### 3.1. Stem Cells from the Dental Pulp Share Similar Morphological Features with Pericytes

**Figure 1:**
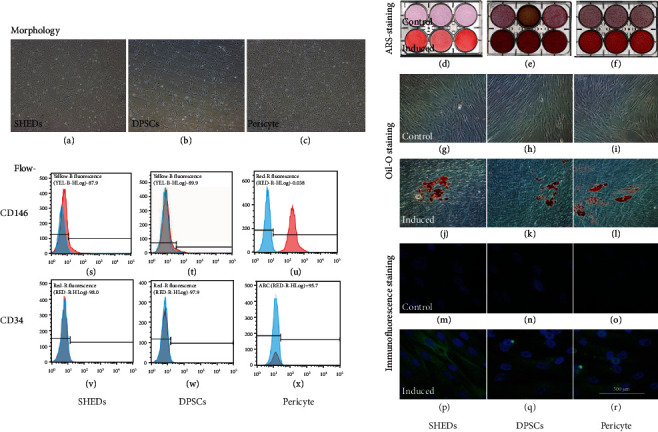
Cell culture and identification. (a–c) pericytes, postnatal human dental pulp stem cells (DPSCs), and stem cells from human exfoliated deciduous teeth (SHEDs) showed similar morphological features under an inverted microscope. (d–f) Alizarin-red staining assay. (g–l) Oil red O staining assay. (m–r) Neurogenic differentiation assay.

**Figure 2:**
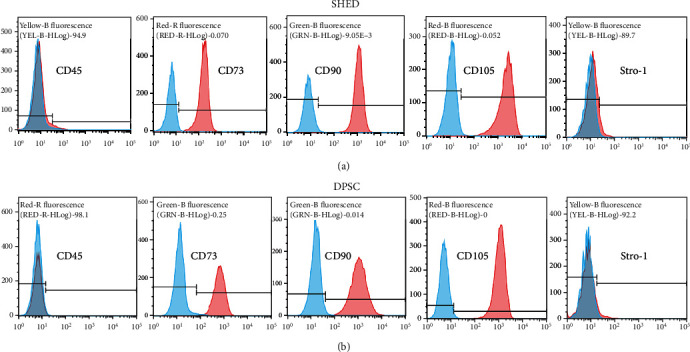
Characterization of cell surface markers of stem cells from human exfoliated deciduous teeth (SHEDs) and postnatal human dental pulp stem cells (DPSCs). Flow cytometry results for the detection of mesenchymal stem cell markers CD45, CD73, CD90, CD105, and STRO-1 of (a) SHED and (b) DPSC.

The DPSCs, SHEDs, and pericytes showed similar morphological features under an inverted microscope (Figures 1(a)–1(c)). Alizarin red staining, Oil red O staining, and immunocytochemistry detected mineralization (Figures 1(d)–1(f)), lipid droplets (Figures 1(g)–1(l)), and the neurogenic marker *β*III-tubulin (Figures 1(m)–1(r)) after induction in osteogenic media, adipogenic media, and neurogenic induction media, respectively. Flow cytometric analyses revealed high expression levels of CD146 in pericytes, but moderate expression levels of CD146 in SHED (+12.1%) and DPSC (+9.07%) (Figures 1(s)–1(u)). However, the expression levels of CD34 were low in these three cell types (Figures 1(v)–1(x)). Flow cytometric analyses revealed high expression levels of CD73, CD90, and CD105, but low expression levels of CD45 in SHED and DPSC; 10.3% of the SHED and 7.7% of the DPSC population expressed Stro-1 (Figures 2(a) and 2(b)).

### 3.2. DPSCs, SHEDs, and Pericytes Stabilized the Vessel-Like Structures Formation of ECs in the Cocultured System

**Figure 3:**
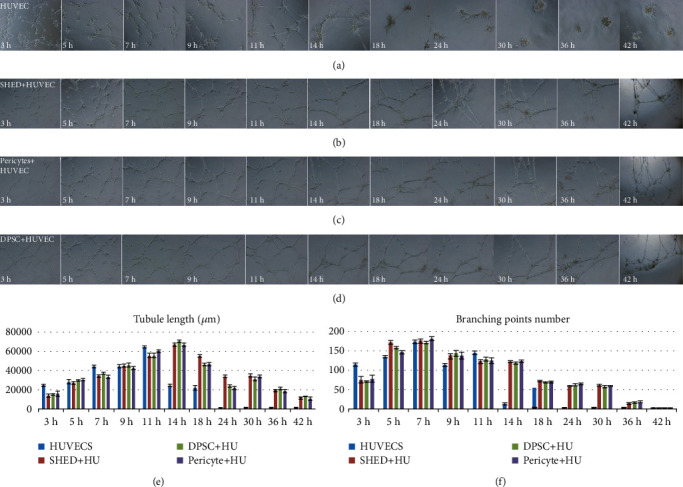
The vessel-like structure formation of endothelial cells (ECs) in coculture. (a) Human umbilical vein endothelial cell (HUVEC) alone. (b) Postnatal human dental pulp stem cell (DPSC)+HUVEC (1 : 1). (c) Stem cell from human exfoliated deciduous teeth(SHED)+HUVEC (1 : 1) were cocultured on Matrigel. (e) Tubule length. (f) Branching point number.

To determine whether DPSCs and SHEDs cocultured with ECs could stabilize established immature vessel-like structures, HUVECs alone, DPSC+HUVEC (1 : 1), and SHED+HUVEC (1 : 1) were cocultured on Matrigel, and vessel-like structures were observed at 3, 5, 7, 9, 11, 14, 18, 24, 30, 36, and 42 h. All of them showed the formation of vessel-like structures in Matrigel at early time points, but the HUVEC alone group displayed degradation after 14 h of incubation, and the other two coculture groups displayed better organized and more developed vessel-like structures at the same time, which indicates that DPSCs and SHEDs played a role similar to that of pericytes in the coculture environment to stabilize immature vessel-like structures (Figure 3).

### 3.3. Migration

**Figure 4:**
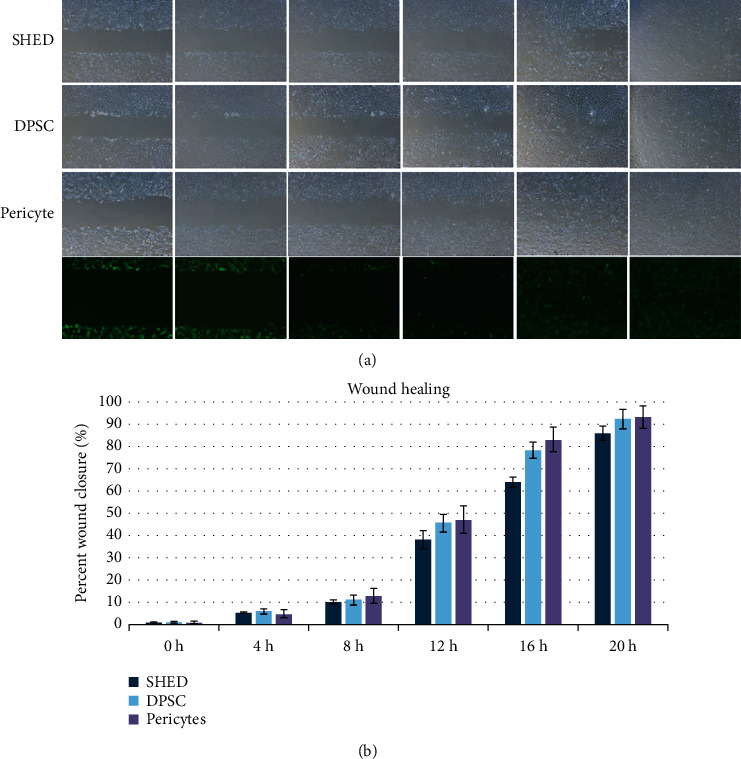
Migration potential of stem cells from human exfoliated deciduous teeth (SHEDs), postnatal human dental pulp stem cells (DPSCs), and pericytes via a wound-healing assay. (a) Phase-contrast images. (b) Percent wound closure (%).

**Figure 5:**
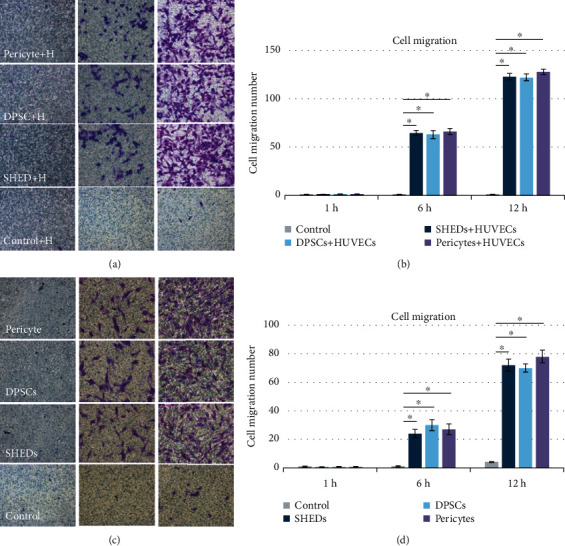
(a, b) The chemotactic effects of stem cells from human exfoliated deciduous teeth (SHEDs), postnatal human dental pulp stem cells (DPSCs), and pericytes on human umbilical vein endothelial cells (HUVECs). (c, d) The migration capacities of SHEDs, DPSCs, and pericytes.

The DPSCs, SHEDs, and pericytes displayed strong migration abilities in the wound healing and transwell assays. Each group showed approximately 10% wound closure after 8 h of incubation and reached 90% healing after 20 h. Statistical analysis showed that there were no significant differences between the groups (*p* > 0.05) (Figures 4(a) and 4(b)). This demonstrated that DPSCs, HUVECs, and pericytes have similar migration abilities. To detect the chemotactic effects of SHEDs, DPSCs, and pericytes on HUVECs, a transwell assay was performed; after 12 h of incubation, the average number of cells was approximately 65 cells at 6 h and 120 cells at 12 h. No significant differences were observed between the groups (Figures 5(a)–5(d)) (*p* > 0.05).

### 3.4. Pericyte mRNA Expression

**Figure 6:**
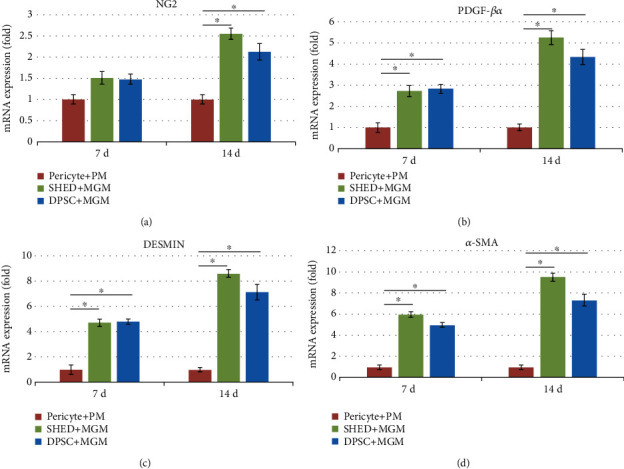
The expression of pericyte-specific cell surface markers after culture in Pericyte Medium (PM) and Melanocyte Growth Medium (MGM)for 14 d. (a) Stem cells from human exfoliated deciduous teeth (SHEDs) (NG2+, 23.3%; DESMIN+, 83.7%; PDGFR-*β*, 93.3%; *α*-SMA, 93.3%). (b) Pericytes (NG2+,74.3%; DESMIN+,67.8%; PDGF-*β*, 85.6%; *α*-SMA, 92.3%). (c) Postnatal human dental pulp stem cells (DPSCs) (NG2+,15.1%; DESMIN+, 58.4%; PDGFR-*β*, 80.6%; *α*-SMA, 91.4%).

Real-time PCR results showed that PDGFR-*β*, *α*-SMA, NG2, and DEMSIN mRNA expression levels in the SHEDs and DPSCs were higher than those in the pericytes at 7 or 14 d of culture with EC medium (Figures 6(a)–6(d)). These genes are pericyte-specific markers, and the results indicate that SHED and DPSC could differentiate into pericytes after 7 or 14 d of induction with EC medium.

### 3.5. Pericyteprotein Expression

**Figure 7:**
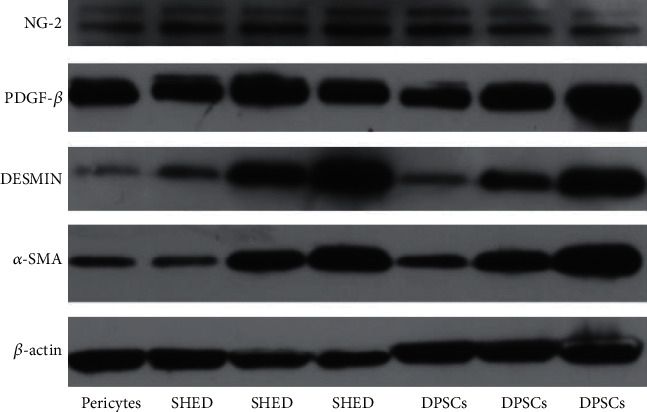
Real-time PCR results of (a) NG2, (b) *α*-SMA, (c) PDGFR-*β*, and (d) DESMIN mRNA expression levels in stem cells from human exfoliated deciduous teeth (SHEDs), postnatal human dental pulp stem cells (DPSCs), and pericytes after culture in Pericyte Medium (PM), *α*-Minimum Essential Medium (MEM), and Melanocyte Growth Medium (MGM) media for 14 d.

Western blot results showed that PDGFR-*β*, *α*-SMA, NG2, and DEMSIN protein expression levels in SHEDs and DPSCs cultured in EC medium were significantly higher than those in the control groups (Figure 7). These results coincided with those of the mRNA expression.

### 3.6. IF Assay

**Figure 8:**
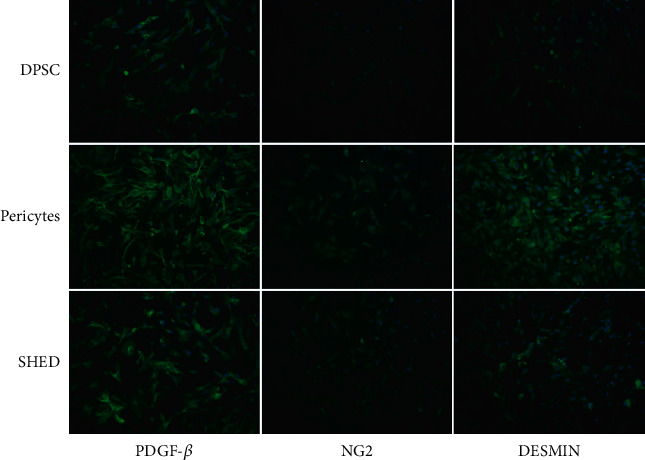
Western blot results of PDGFR-*β*, *α*-SMA, NG2, and DESMIN protein expression levels in SHED, DPSC, and pericytes cultured in PM, *α*-MEM, and MGM medium for 14 days.

To assess the time-dependent effects of Melanocyte Growth Medium (MGM) on SHED and DPSC differentiation, we analyzed the protein expression levels of pericyte-specific markers on day 14. The results showed that the pericyte-specific markers of PDGFR-*β*, *α*-SMA, NG2, and DESMIN in SHEDs and DPSCs cultured in MGM medium were significantly higher than those in the control groups (Figure 8).

### 3.7. Flow Cytometry Analysis

**Figure 9:**
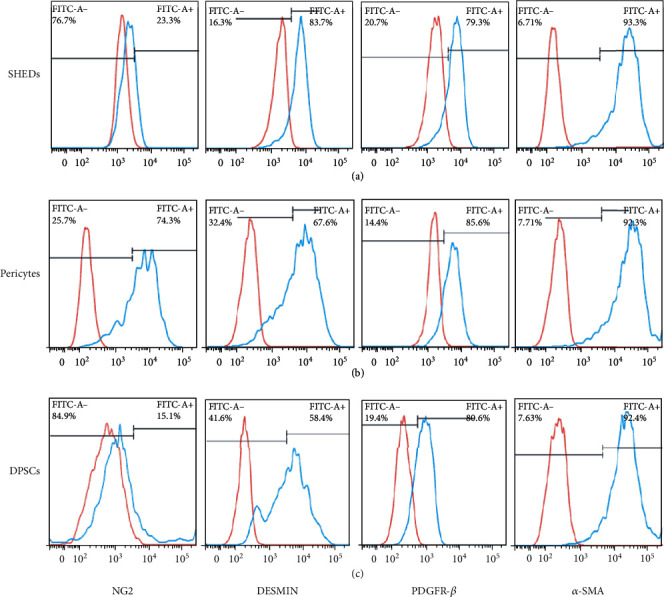
Immunofluorescence staining of pericyte-specific markers of PDGFR-*β*, NG2, and DESMIN in stem cells from human exfoliated deciduous teeth (SHEDs), postnatal human dental pulp stem cells (DPSCs), and pericytes cultured in Melanocyte Growth Medium (MGM).

As shown in Figure 9, a high proportion of cells expressed pericyte markers on day 14 in SHEDs (NG2+, 23.3%; DESMIN+, 83.7%; PDGFR-*β*+, 93.3%; *α*-SMA, 93.3%), pericytes (NG2+, 74.3%; DESMIN+, 67.8%; PDGFR-*β*+, 85.6%; *α*-SMA+, 92.3%), and DPSCs (NG2+, 15.1%; DESMIN+, 58.4%; PDGFR-*β*+, 80.6%; *α*-SMA+, 91.4%).

## 4. Discussion

The SHEDs and DPSCs are highly proliferative, clonogenic cells capable of differentiating into a variety of cell types, including neural cells, adipocytes, and odontoblasts. However, very few studies have investigated their potential to promote the functional recovery of blood vessels [10, 19]. Previous studies have demonstrated that SHEDs and DPSCs are potential candidate cell sources for endothelial differentiation, and the canonical Wnt/*β*-catenin pathway is involved in this process [20]. Stem cells from human exfoliated deciduous teeth possess a higher endothelial differentiation potential than DPSCs [21, 22]. Such induced endothelial cells only partially express some surface markers or proteins and have a very limited ability to form blood vessels [21]. In addition, previous studies have shown that both DPSCs and SHEDs can be successfully induced into functional SMCs [23, 24]. Comparative studies have indicated that craniofacial pericytes and DPSCs originate from the cephalic neural crest [25]. The anatomical position and developmental origin of DPSCs are similar to those of pericytes [14]. However, the capacity of SHEDs to function as pericytes has not yet been elucidated. In this study, the stemness, pericyte-like functions, and capacity to induce angiogenesis in SHEDs, DPSCs, and pericytes were compared systematically. We compared the characteristics of DPSCs, SHEDs, and pericytes that showed similar morphological features under an inverted microscope. Flow cytometric analyses revealed that the SHEDs, DPSCs, and pericytes expressed CD146, but not CD31, which is a widely known characteristic of pericyte surface markers [26]. Alizarin red staining, Oil red O staining, and immunocytochemistry also detected mineralization and lipid droplets after induction in osteogenic and adipogenic media, respectively. Promoting tissue angiogenesis is the first step in tissue engineering and stem cell therapy [27]. How to form functional blood vessels and establish stable and effective microcirculation are important issues [28]. A previous study indicated that pericyte-induced angiogenic processes include several steps.

In the first step, pericytes secrete angiogenic factors (e.g., vascular endothelial growth factor [VEGF]) to promote endothelial cell migration toward the factor concentration gradient. In the second step, pericytes stabilize the migration of endothelial cells and generate vascular-like structures [29].

To investigate the effects of SHEDs, DPSCs, and pericytes on the migration of HUVECs, transwell assays were performed. Stem cells from human exfoliated deciduous teeth, DPSCs, and pericytes had strong chemotactic effects on HUVECs, and there were no significant differences among the three cell types. In addition, SHEDs, DPSCs, and pericytes exhibited strong migration capacities.

To investigate the effect of SHEDs, DPSCs, and pericytes on stabilizing vascular-like structures formed by HUVECs, HUVECs were seeded on Matrigel alone or together with SHEDs, DPSCs, or pericytes. The branching point number and tubule length were observed from 3 to 70 h. Vascular-like structures formed by HUVECs alone began to degenerate at 14 h, whereas the vascular-like structures of cocultures persisted for 30 h before degeneration. Pericytes express CD31-/CD34+ surface markers, but the expression patterns of other markers such as DESMIN, *α*-SMA, NG2, PDGFR-*β*, CD146, and CD144 can vary in a tissue-specific manner or depend on the developmental or angiogenic stage of a blood vessel [30].

To further evaluate the characteristics and phenotypes, immunocytochemistry, RT-PCR, flow cytometry, and western blotting were performed. We cultured SHEDs and DPSCs in PM, *α*-MEM, and MGM, respectively. After 7 d of culture, the mRNA and protein expression of DESMIN and *α*-SMA in the SHEDs and DPSCs increased significantly, while NG2 and PDGFR-*β* were strongly expressed consistently.

Flow cytometry showed that high proportions of SHEDs, DPSCs, and pericytes expressed *α*-SMA, PDGFR-*β*, and DESMIN markers (*α*-SMA+, 93.3%, 92.3%, and 92.4%; PDGFR-*β*+79.3%, 80.6%, and 85.6%; DESMIN+, 83.7%, +58.4%, and 67.6%, respectively), moderate proportions of the SHEDs and DPSCs positively expressed NG2 (23.3% and 15.1%, respectively). These results indicate that SHEDs and DPSCs possess characteristics consistent with muscle cell activity and express contractile smooth muscle actin, which is considered a major feature of pericytes [2]. Additionally, immunocytochemistry was performed on day 7, which supported the western blotting and flow cytometry data.

## 5. Conclusion

Our data indicate that SHEDs and DPSCs share similar functions with pericytes. This study provides a solid theoretical foundation for the clinical use of dental pulp stem cells as a potential candidate to replace pericytes.

## Figures and Tables

**Table 1:** Primer sequences.

Human gene	Primers
*NG2*	Fw: AGTGTGGTGGACCCAGACTC
	Rev: CATTGACACCCCTAGCCAGT
*α-SMA*	Fw: CCGACCGAATGCAGAAGGA
	Rev: ACAGAGTATTTGCGCTCCGAA
*PDGF-β*	Fw: AATGTCTCCAGCACCTTCGT
	Rev: AGCGGATGTGGTAAGGCATA
*DESMIN*	Fw: GCTGAAAGAAGAAGCGGAGAAC
	Rev: GAGCTAGAGTGGCTGCATCCA
*GAPDH*	Fw: TGCACCACCAACTGCTTAGC
	Rev: GGCATGGACTGTGGTCATGAG

## Data Availability

The data used to support the findings of this study are available from the corresponding authors upon request.
